# Applicability of a Monolithic Column for Separation of Isoquinoline Alkalodis from *Chelidonium majus* Extract

**DOI:** 10.3390/molecules24193612

**Published:** 2019-10-07

**Authors:** Michał Staniak, Magdalena Wójciak-Kosior, Ireneusz Sowa, Maciej Strzemski, Jan Sawicki, Sławomir Dresler, Katarzyna Tyszczuk-Rotko

**Affiliations:** 1 Department of Analytical Chemistry, Medical University of Lublin, 20-093 Lublin, Poland; michal_staniak@wp.pl (M.S.); i.sowa@umlub.pl (I.S.); maciej.strzemski@poczta.onet.pl (M.S.); jan.sawicki@mgr.farm (J.S.); 2 Department of Plant Physiology and Biophysics, Maria Curie-Skłodowska University, 20-033 Lublin, Poland; dresler.slawomir@gmail.com; 3 Faculty of Chemistry, Maria Curie-Skłodowska University, 20-031 Lublin, Poland; ktyszczuk@poczta.umcs.lublin.pl

**Keywords:** Alkaloids, isoquinoline alkaloids, monolithic columns, *Chelidonium majus*, HPLC

## Abstract

Isoquinoline alkaloids are the main group of secondary metabolites present in *Chelidonium majus* extracts, and they are still the object of interest of many researchers. Therefore, the development of methods for the investigation and separation of the alkaloids is still an important task. In this work, the application potential of a silica-based monolithic column for the separation of alkaloids was assessed. The influence of the organic modifier, temperature, salt concentration, and pH of the eluent on basic chromatographic parameters such as retention, resolution between neighboring peaks, chromatographic plate numbers, and peak asymmetry were investigated. Based on the obtained results, a gradient elution program was developed and used to separate and quantitatively determine the main alkaloids in a *Chelidonium majus* root extract.

## 1. Introduction

Alkaloids are a highly diverse group of compounds that naturally occur in the plant kingdom [1,2]. There are many definitions describing this group; however, from an analytical point of view, the most important feature of alkaloids is their basic character resulting from the presence of a heterocyclic nitrogen atom [3].

In this group of compounds, isoquinoline alkaloids represent a large subgroup characterized by the occurrence of an isoquinoline or tetrahydroisoquinoline ring in the molecule [4,5]. They have been a subject of interest in numerous investigations, as they have various biological activities including antibacterial, antiviral, antifungal, and antitumor effects [6,7]. Plants containing high levels of isoquinoline alkaloids, e.g., *Chelidonium majus*, have been widely used in traditional folk medicine as a remedy for eye diseases, ulcers, skin eczema, and gastric problems such as colic and jaundice [8].

Despite the development of different alternative approaches for obtaining alkaloids, such as microbial engineering or chemical synthesis, plants are still the main sources of these compounds. Therefore, the development of methods for the investigation and separation of the alkaloids is still an important task. Nowadays, reversed phase high performance liquid chromatography (RP HPLC) with the use of columns with spherical filling is the most common technique applied in analysis of plant material [9]. This technique ensures the precision, accuracy, and repeatability of determination; additionally, this type of adsorbent has great separation efficiency. However, its main drawback is its susceptibility to clogging due to the presence of a rich plant matrix, which can result in a significantly shortened lifetime of the column and a reduced repeatability of analyses. Moreover, other limitations include the large flow resistance, high back pressure, and relatively low mobile phase flow rate [10].

As shown by literature data [11,12], the replacement of these types of columns with monolithic beds may reduce their problems. Silica monolithic columns, also called “silica rods” [13], consist of one piece of continuous, porous, and rigid structure of silica [14]. Due to their porous structure, they have a much higher permeability to the mobile phase, and, hence, high flow rates can be applied with the maintenance of acceptable back pressure values. The ability to connect several monolithic columns increases the efficiency of this chromatographic system, which is particularly beneficial in the case of analyzing samples with a complex matrix or compounds with a similar structure [15].

Commercially available monolithic columns have previously been applied in the analysis of various plant metabolites such as flavonoids [16,17,18,19], phenolic acids [20,21,22], and furocoumarins [23]. However, there are currently only few papers on their application of HPLC to alkaloids. For instance, Srivastava et al. used a monolithic column to separate reserpine, ajmaline, and ajmalicine in *Rauvolfia serpentine,* and Sparzak et al. analyzed securinega-type alkaloids from *Phyllanthus glaucus* [24,25].

The aim of our work was to investigate the application potential of commercially available silica-based monolithic beds to separate alkaloids. *Chelidonium majus*, which is a rich source of isoquinoline alkaloids, was chosen as a plant model. The step-by-step optimization of the procedure was performed using different mobile phase compositions with a simultaneous assessment of basic chromatographic parameters such as retention, resolution between neighboring peaks, chromatographic plate numbers, and peak asymmetry.

## 2. Results and Discussion

Depending on pH, alkaloids may exist as free bases or as cationic forms; this results in the occurrence of the dual retention mechanism (RP and the ion-exchange retention mechanism) on the RP-type stationary phase. Therefore, the chromatographic peaks of alkaloids are often wide, asymmetric, and tailed, which impede their separation and proper integration for quantitative purposes. Thus, the choice of optimal working parameters for HPLC such as mobile phase composition and the type of adsorbent is the first most important step of chromatographic investigation. Some key factors, e.g., the resolution between neighboring peaks (R_s_), the symmetry of peaks (A_s_), and system efficiency expressed as a theoretical plate number (N), must be taken into consideration.

In our work, a column with monolithic filling was tested for its suitability in the HPLC of isoquinoline alkaloids. The impact of three variables—the concentration of the organic modifier, the concentration of ammonium acetate, the pH value of the eluent—on the chromatographic parameters for acetonitrile/water solvent systems was investigated.

### 2.1. Concentration of Ammonium Acetate and pH of the Mobile Phase

Anionic ion-pairing reagents such as salts and an adequate pH of the mobile phase are used for a minimization of the interaction of a base compound with the stationary phase to avoid tailing and asymmetric peaks. Therefore, the initial influence of salt concentrations (10, 15, and 20 mM) and pH (values of 3, 4, 5) on the investigated parameters were tested by performing cross trials. The content of the organic modifier was constant at 20% of acetonitrile.

Our investigation showed that alkaloids were eluted as follows: Protopine (prot), allocryptopine (allo), chelidonine (cheli), coptisine (copt), sanguinarine (sang), berberine (berb), and chelerythrine (chele) in most of the mobile phase compositions; however, the order at pH 5 was changed, i.e., coptisine was eluted before chelidonine (Figure S1).

**Figure 1:**
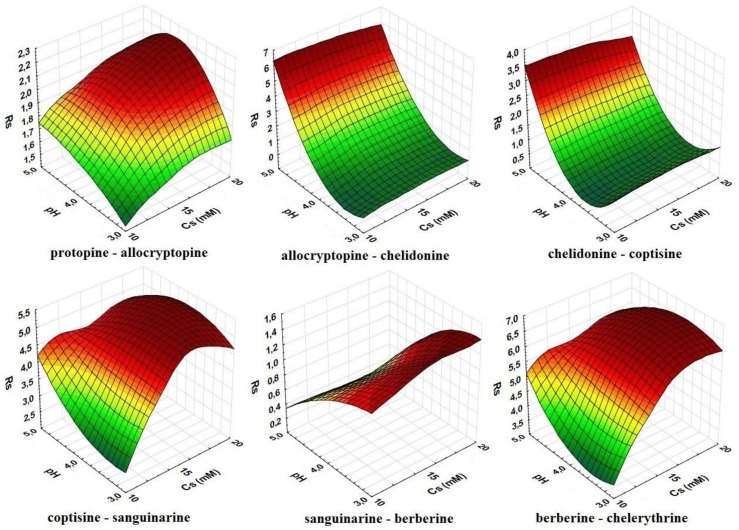
Relationship between the resolution (R_s_) of neighboring peaks on the chromatogram and pH of the eluent and ammonium acetate concentrations in the mobile phase, which was composed of water and acetonitrile (8:2, *v*/*v*).

The relationship between two investigated variables and the resolution of neighboring peaks on the chromatogram are presented in Figure 1.

The resolution between allocryptopine and chelidonine, between chelidonine and coptisine, and between sanguinarine and berberine depended mostly on the pH value. At higher values, the resolution was improved for allo–cheli and cheli–copt, whereas sanguinarine and berberine were better separated at a low pH. In contrast, the salt concentration strongly affected the separation of berb–chele, copt–sang, and prot–allo, and the R_s_ value increased at the higher content of ammonium acetate; pH only had a slight impact on the resolution of these pairs.

**Figure 2:**
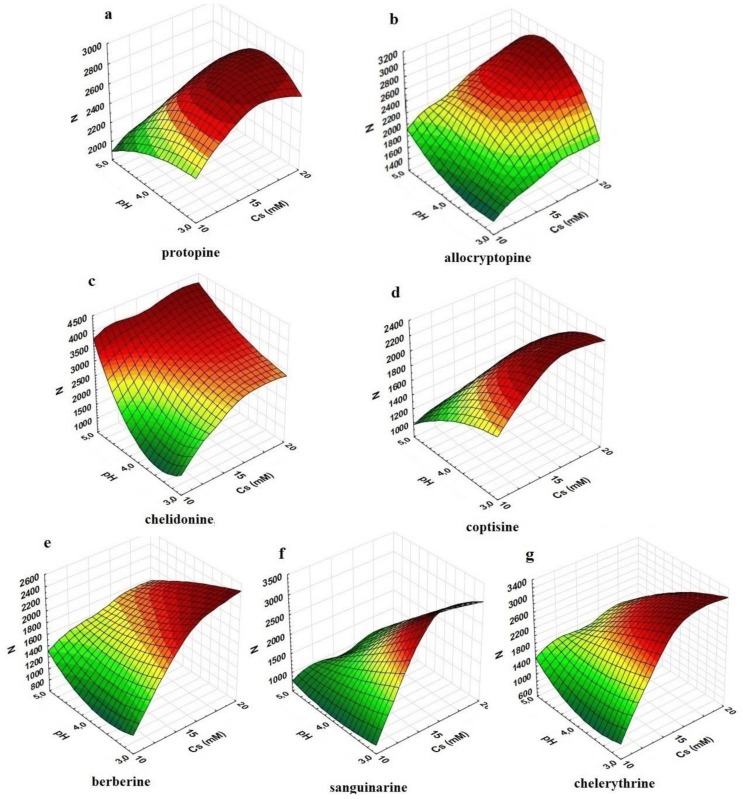
Relationship between the efficiency of the chromatographic system (N), the pH of the eluent, and ammonium acetate concentration in the mobile phase, which was composed of water and acetonitrile (8:2, *v*/*v*): (**a**)—protopine; (**b**)—allocryptopine; (**c**)—chelidonine; (**d**)—coptisine; (**e**)—berberine; (**f**)—sanguinarine; (**g**)—chelerythrine.

The salt concentration and pH of the mobile phase also strongly affected the efficiency of the chromatographic system and the symmetry of peaks. Theoretical plate numbers (N) decreased at the low salt concentration for all compounds and decreased at a low pH for allo and cheli (Figure 2a,b). An opposite effect was noted for the other investigated compounds—the N number was lower at a high pH value (Figure 2c–g).

Considering the asymmetry of the peaks, a similar tendency was observed for all alkaloids. Generally, the peaks tailed (A_s_ > 1,2), and their symmetry was better at the low pH value and at the higher salt content (Figure S2).

### 2.2. Concentration of Acetonitrile

**Figure 3:**
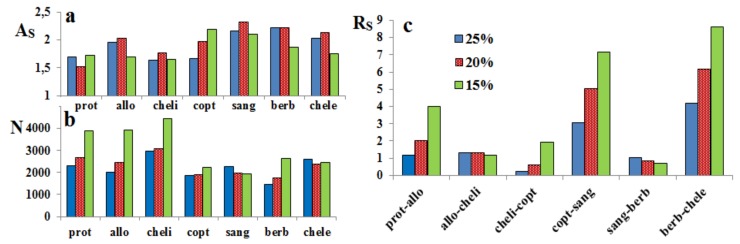
Relationship between the concentration of acetonitrile in the mobile phase and: (**a**) asymmetry (A_s_), (**b**)the efficiency of the chromatographic system (N), and (**c**)—the resolution of neighboring peaks on the chromatogram (R_s_).

In the next step of our investigations, the influence of different concentrations of the organic modifier in the mobile phase on the chromatographic parameters was tested. Methanol and acetonitrile were considered because they are the most common eluents for RP HPLC analysis. The results for the acetonitrile/water mixture are shown in Figure 3.

As can be seen in the figure, the amount of acetonitrile exerted a diverse effect on the peak symmetry and the efficacy of the chromatographic system. For example, the peak of coptisine had better symmetry at the higher concentration; in contrast, the symmetry of berberine, sanguinarine, and allocryptopine was better at the lower concentration of the organic solvent (Figure 3a). The theoretical plate number for prot, allo, cheli, and berb increased with the decreasing amount of the organic solvent, whereas the differences for the other alkaloids were insignificant (Figure 3b).

The resolution between prot–allo, cheli–copt, copt–sang, and berb–chele significantly increased at the low concentration of acetonitrile; only slight differences were observed for allo–cheli and sang–berb.

As in our previous research [26], when methanol was used instead of acetonitrile as an organic modifier, the alkaloid peaks were broadened and had high asymmetry; thus, the efficacy of the chromatographic systems was poor (Table S1, Figure S3). However, the resolution between sanguinarine and berberine was better for the methanol/water system than for the acetonitrile/water mobile phase.

### 2.3. Effect of Temperature

Temperature exerts an effect on chromatographic separation, and a decline in the temperature can improve the resolution of components with a similar chemical structure in some cases. As shown by our results (Figure S4), the changes in the temperature had a minor impact on A_s_, N, and the resolution of alkaloids.

### 2.4. Combination of Two and Three Columns

Monolithic columns can be easily combined to extend the interaction with the adsorbent and thus enhance the separation efficiency. Table 1 presents the chromatographic parameters obtained using one and a combination of two and three columns.

**Figure 4:**
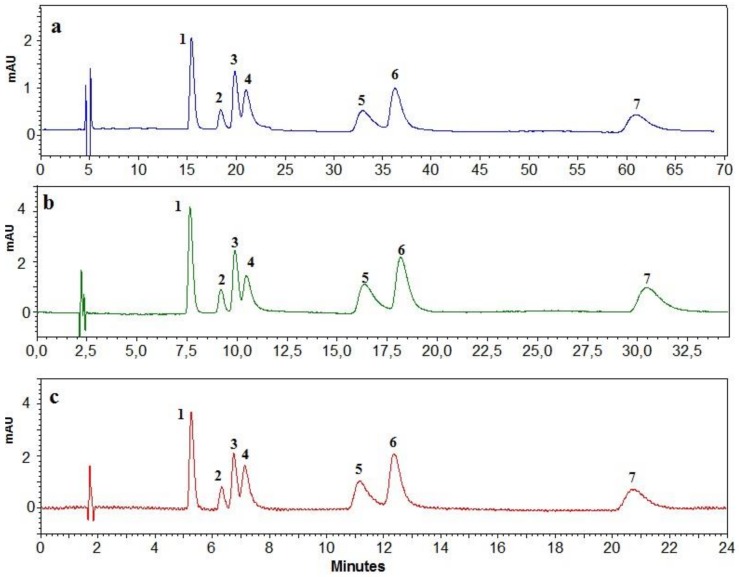
Example of a chromatogram obtained using three monolithic columns and a flow rate of 1(**a**), 2(**b**) and 3(**c**) mL/min. Mobile phase composed of acetonitrile and a 15 mM aqueous solution of ammonium acetate adjusted with acetic acid to pH 4 (2:8, v/v): 1—protopine; 2—allocryptopine; 3—chelidonine; 4—coptisine; 5—sanguinarine; 6—berberine; and 7—chelerythrine.

As expected, the retention times increased proportionally to the length of the chromatographic bed, and the resolution between the peaks was improved. The R_S_ value for a majority of compounds was higher than 1.5 when a combination of three columns was used; only chelidonine and coptisine were still not separated to the base line. In the next step, the influence of an increased flow rate on separation efficiency was investigated. Due to low back pressure, the flow rate of the eluent during chromatography on monolithic beds may be higher than for spherical columns. This increased flow rate avoids a prolongation of the time of the chromatographic run. Figure 4 presents the chromatographic separation of isoquinoline alkaloids with the use of a combination of three columns at a flow rate of the mobile phase of 1, 2 and 3 mL/min.

### 2.5. Analysis of Plant Material

Based on the obtained results, the elution gradient program was developed to separate alkaloids present in the *C. majus* root extract. The optimal conditions were established as follows: Three monolithic columns, a mobile phase consisting of acetonitrile (A), a 15 mM water solution of ammonium acetate adjusted to a pH of 4 with acetic acid (B), and methanol (C). The flow rate was 2 mL/min, and the temperature of the thermostat was 25 °C. The elution gradient program was as follows: From 0 to 20 min—15% A, 3% C, and 82% B; from 20.5 to 40.0 min—40% C and 60% B. As can be seen, in a gradient elution, methanol was added to increase the separation between sanguinarine and berberine.

**Figure 5:**
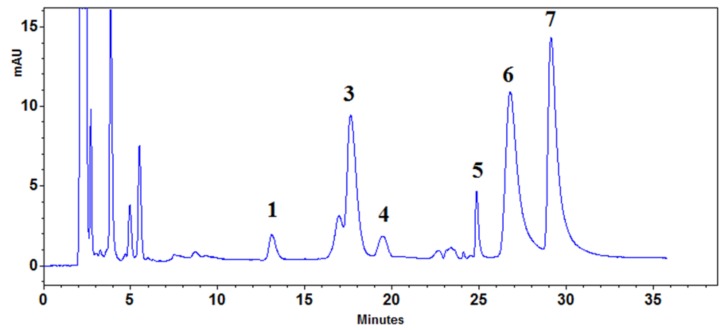
Example of 2D chromatograms obtained using a combination of three monolithic columns and the developed gradient elution program: 1—protopine; 3—chelidonine; 4—coptisine; 5—berberine; 6—sanguinarine; and 7—chelerythrine.

The results of the chromatographic separation are shown in Figure 5 and Figure S5, and the results of the quantitative analysis of the main alkaloids found in the plant extract are summarized in Table S2.

Spherical RP-type columns are most often applied to quantify isoquinoline alkaloids from *Chelidonium majus* using the HPLC method [27]. Though there have been many papers describing the separation of alkaloids [28,29,30], most of them have not presented chromatographic data. In some papers, the authors have only shown chromatograms and used retention times as the main chromatographic parameter. For instance, Petruczynik et al. [31] described an efficient chromatographic system to separate protopine, chelidonine, berberine, sanguinarine, and chelerythrine in a *C. majus* extract for 45 min on an RP-8 spherical filling with a mobile phase containing 28% acetonitrile, water and 0.04 M/L, 1-butyl-3-methylimidazolium tetrafluoroborate. Kursinszki et al. [30] separated protopine, chelidonine, coptisine, berberine, and sanguinarine for 34 min on an RP-18 column with the use of a mobile phase composed of acetonitrile–methanol–30 mM ammonium formate 14.7:18:67.3 (v/v/v); however, chelerythrine, which has the strongest interaction with the stationary phase, was not included to the study. In chromatographic systems proposed by researchers, coelution within the investigated analytes was often observed [32].

There has only been one systematic study describing the impact of mobile phase composition on the chromatographic parameters of isoquinoline alkaloids separated with the use of an XB-C18 core-shell column. The conditions proposed by Sowa et al. [26] allowed for the separation of alkaloids to the base line within 40 min, and the chromatographic system had a high efficacy (a high theoretical plate number), which is typical for the core-shell column. However, this type of adsorbent is highly susceptible to clogging when the analyzed samples, such as plant extracts, have a rich matrix. The regeneration of the core-shell column before a subsequent injection is relatively long and requires a large amount of organic solvents to clean up the adsorbed matrix. In contrast, because of the unique porous structure of monolithic beds, sample components are much more easily eluted due to the multiple pathways of mobile phase dispersion. Moreover, the low back pressure of the monolithic column allows for the application of a higher flow rate; thus, the time of regeneration is reduced [10].

Through our study, an efficient chromatographic system for the separation of isoquinoline alkaloids from a *C. majus* root extract with the use of monolithic column was elaborated, and it turned out to be an attractive alternative to commonly applied spherical columns.

## 3. Materials and Methods

### 3.1. Chemicals and Reagents

Alkaloid standards protopine, allocryptopine, berberine, chelidonine, chelerythrine, and sanguinarine were purchased from Sigma (St. Louis, MO, USA), and coptisine was purchased from ChromaDex (Los Angeles, CA, USA). Ammonium acetate, acetic acid, HPLC-grade methanol, and acetonitrile were supplied by Merck (Darmstadt, Germany). The liquid chromatography LC grade water was prepared using an ULTRAPURE Millipore Direct-QVR 3UV-R system (Merck, Darmstadt, Germany).

### 3.2. Chromatographic Experiments

The HPLC analysis was performed with a VWR Hitachi Chromaster 600 chromatograph with a spectrophotometric detector (DAD) on a Chromolith^®^ Performance RP-18e column (100 × 4.6 mm) (Merck, Darmstadt, Germany). The chromatographic parameters were evaluated using EZChrom Elite Software (Merck, Darmstadt, Germany). All chromatographic experiments were replicate tree times. Figures presenting interaction between variables were created using Statistica 10 software (StatSoft, Polska).

### 3.3. Plant Material

*Chelidonium majus* was collected in Lublin in May 2019. The plant material was frozen at −60 °C and freeze-dried in ALPHA 2-4 LDplus (Christ, Germany).

### 3.4. Sample Preparation

The extraction of roots (50.13 mg) from *Chelidonium majus* was performed in Eppendorf tubes in an ultrasonic bath (4 × 15 min). Samples were extracted with 1 ml of methanol containing 0.05 M hydrochloric acid. The extracts were separated from the plant powder by centrifugation. Four extracts were combined and supplemented with methanol containing 0.05 M hydrochloric acid to a volume of 10 mL in a volumetric flask.

### 3.5. Quantitative HPLC Analysis

Three Chromolith^®^ Performance RP-18e (Merck, Darmstadt, Germany) (100 × 4.6 mm) columns were used for chromatographic separations. Chromatograms were recorded in the wavelength range of 220–400 nm. The HPLC conditions were established as follows: Three monolithic columns, a mobile phase consisting of acetonitrile (A), a 15 mM water solution of ammonium acetate adjusted to a pH of 4 with acetic acid (B), and methanol (C). The flow rate was 2 mL/min, and the temperature of the thermostat was 25 °C. The gradient elution program was as follows: From 0 to 20min—15% A, 3% C, and 82% B; from 20.5 to 40.0 min—40% C and 60% B. The identities of the compounds in the plant extracts were confirmed by a comparison of retention times and spectra with corresponding standards.

## Figures and Tables

**Table 1:** Retention times (t_R_), theoretical plate number (N), and resolution between neighboring peaks (R_s_) obtained for the mobile phase composed of 20% acetonitrile and a 15 mM aqueous solution of ammonium acetate adjusted, using acetic acid, to pH 4 with the use of one and a combination of two and three monolithic columns.

	One Column			Two Columns			Three Columns		
	t_R_	N	R_S_	t_R_	N	R_S_	t_R_	N	R_S_
**Protopine**	4.91	2695	2.05	9.893	3547	2.84	15.047	5650	3.50
**Allocryptopine**	5.75	2471	1.32	11.6	3821	1.43	18.04	6339	1.57
**Chelidonine**	6.34	3105	0.62	12.76	3582	0.81	19.5	5893	0.97
**Coptisine**	6.67	1933	5.05	13.347	2250	5.87	20.607	3777	6.16
**Sanguinarine**	10.4	2001	0.88	20.807	2114	1.31	32.36	2693	1.51
**Berberine**	11.2	1765	6.21	23,14	2979	7.65	35.847	4437	8.17
**Chelerythrine**	19.2	2418		38.687	2643		60.42	3726	

## Supplementary Materials

The supplementary materials are available online.
