# Supplementary material for: Applicability of a Monolithic Column for Separation of Isoquinoline Alkalodis from Chelidonium majus Extract
Source: Molecules. 2019 Oct 7;24(19):3612. doi: 10.3390/molecules24193612 (PMC6804227; doi:10.3390/molecules24193612)
Supplement: Supplementary file 1 [file molecules-24-03612-s001.pdf]

Applicability of a Monolithic Column for Separation of Isoquinoline Alkalodis from *Chelidonium majus* Extract

Michał Staniak[1], Magdalena Wójciak-Kosior[1,*], Ireneusz Sowa[1], Maciej Strzemski[1], Jan Sawicki[1], Sławomir Dresler[2] and Katarzyna Tyszczuk-Rotko[3]

[1] Department of Analytical Chemistry, Medical University of Lublin, Chodźki 4a, 20-093 Lublin, Poland
[2] Department of Plant Physiology, Maria Curie-Skłodowska University, Akademicka 19, 20-033 Lublin, Poland
[3] Faculty of Chemistry, Maria Curie-Skłodowska University, Maria Curie-Skłodowska Square 3, 20-031 Lublin, Poland
* Correspondence: kosiorma@wp.pl

(a)

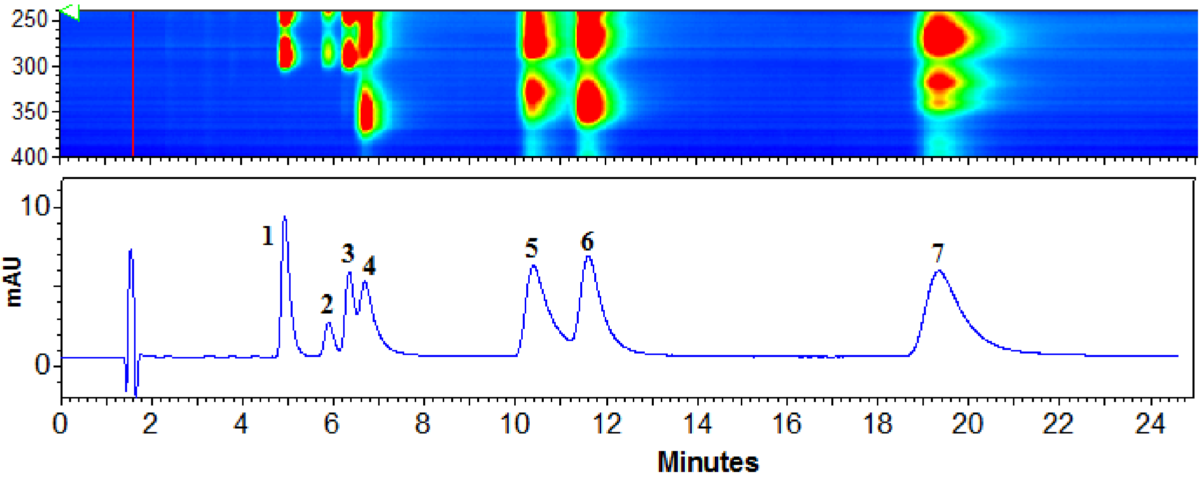

(b)

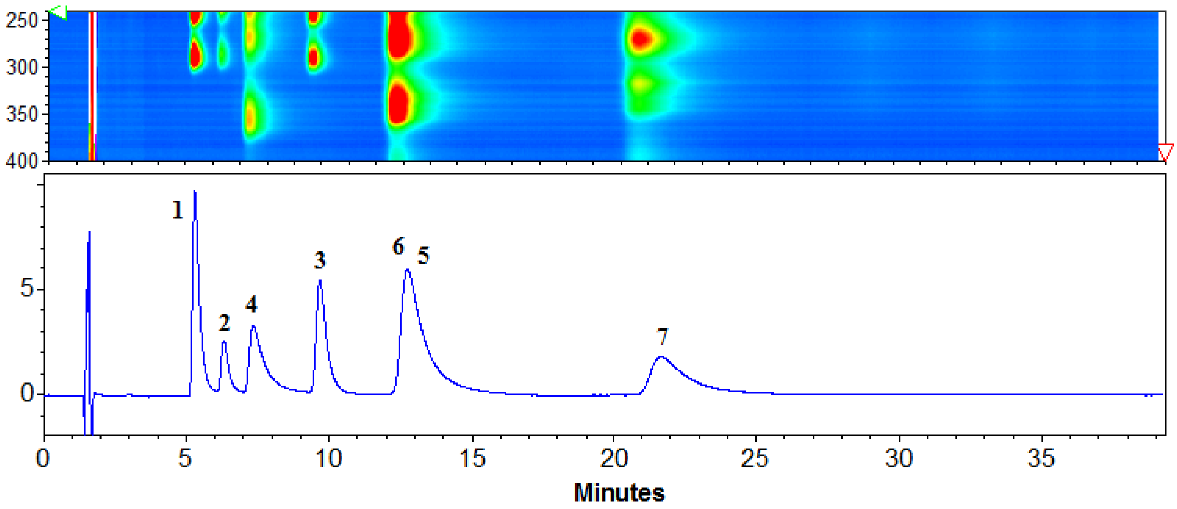

Figure 1S. Example of chromatogram obtained using monolithic column as a stationary phase and mobile phase composed of acetonitrile and 15mM aqueous solution of ammonium acetate (2:8, v/v) adjusted with acetic acid to pH 4 **(a)** and to pH 5 **(b)**: 1- protopine, 2- allocryptopine, 3-chelidonine, 4- coptisine, 5- sanguinarine, 6- berberine, and 7- chelerythrine.

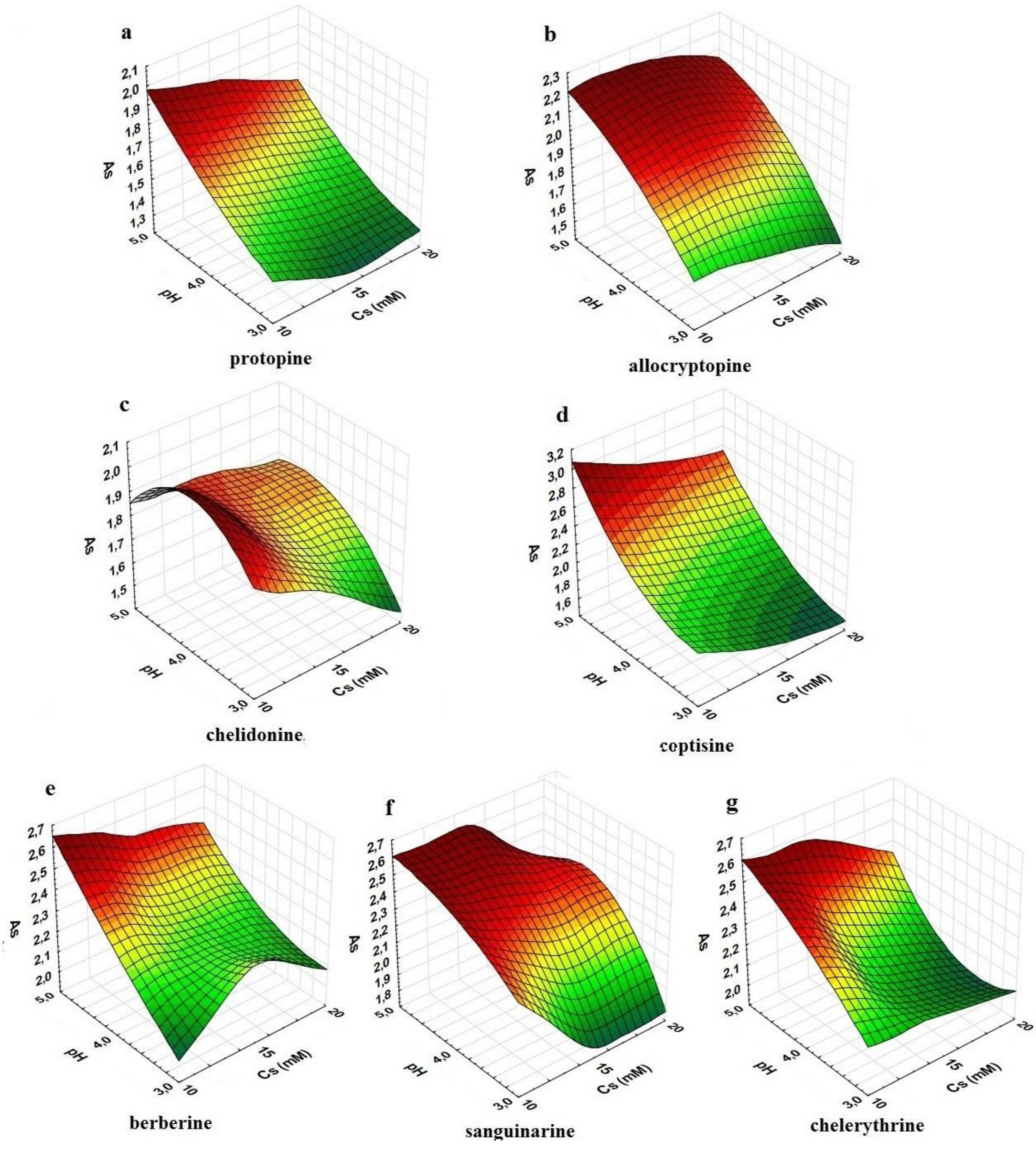

Figure 2S. Relationship between asymmetry (A_S) of chromatographic peaks and pH of eluent and ammonium acetate concentration in mobile phase composed of water and acetonitrile (8:2, v/v).

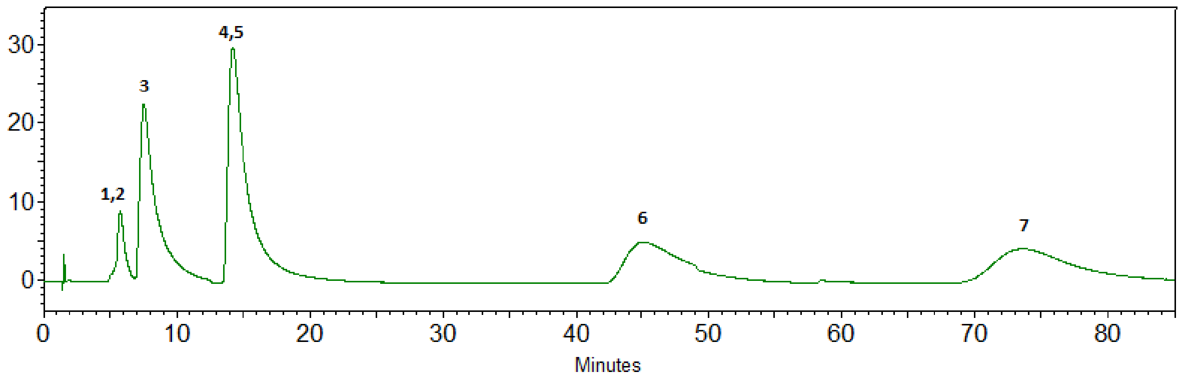

Figure 3S. Example of chromatogram obtained using monolithic column as a stationary phase and mobile phase composed of methanol and 15mM aqueous solution of ammonium acetate (3:7, v/v) adjusted with acetic acid to pH 5. 1- protopine, 2- allocryptopine, 3-coptisine, 4-chelidonine, 5- berberine, 6- chelerythrine, and 7-sanguinarine

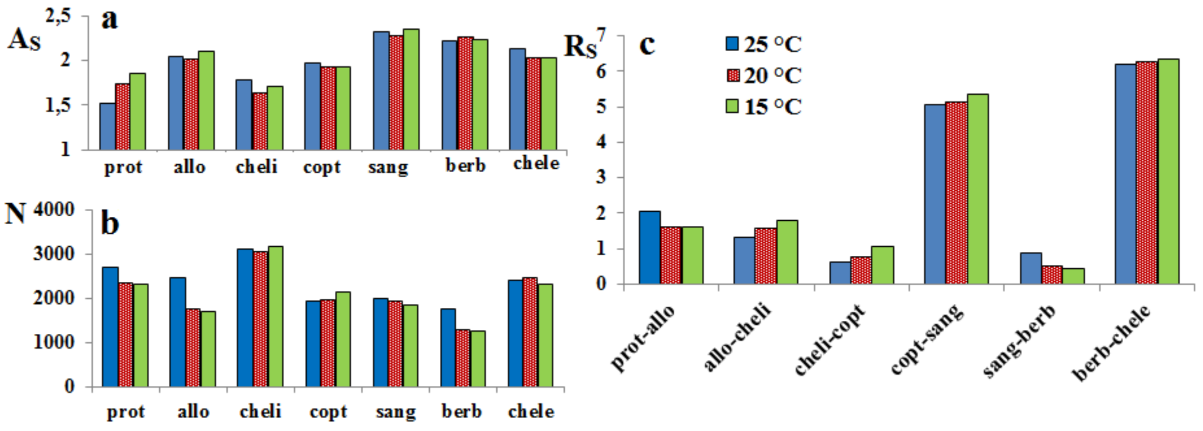

Figure 4S. Relationship between temperature and asymmetry (A_S), efficiency of chromatographic system (N) and resolution of neighbouring peaks on chromatogram (R_S).

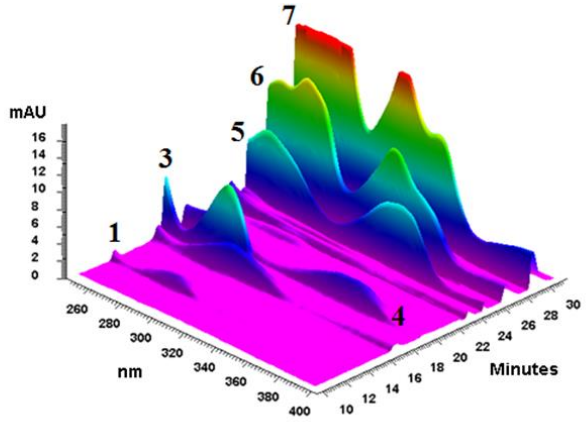

Figure 5S. Example of 3D chromatograms obtained using combination of three monolytic columns and elaborated gradient elution program: 1- protopine, 3-chelidonine, 4- coptisine, 5- berberine, 6- sanguinarine, and 7-chelerythrine.

Table S1. Comparison of system efficiency using methanol and acetonitrile as an organic modifier. Temperature of the column - 25°C, flow rate of the mobile phase - 1ml/min. Abbreviations: N – theoretical plate number, $W_{50\%}$ - width at 50% high.

	30%MeOH 15mM CH_3COONH_4 pH=4		20%ACN 15mM CH_3COONH_4 pH=4	
	N	$W_{50\%}$	N	$W_{50\%}$
protopine	1008	0.43	2695	0.22
allocryptopine	721	0.53	2471	0.26
chelidonine	1466	0.42	3105	0.27
coptisine	541	0.7	1933	0.35
berberine	1045	0.88	1765	0.62
sanguinarine	701	1.73	2001	0.52
chelerythrine	910	2.25	2418	0.91

Table S2. Validation parameters and the results of quantitative analysis of alkaloids in *C. majus* root.

Compound	Curve equation	R^2	LOD (mg/mL)	LOQ (mg/mL)	Content (mg/100g ±SD)
protopine	y = 20 979 870x + 14 372	0.9999	0.00082	0.00245	158.50 ±3.88
allocryptopine	y = 10 079 605 x + 17 065	0.9992	0.00313	0.00939	-
chelidonine	y = 40 221 072x – 22 242	0.9999	0.00249	0.00746	714.54 ±37.35
coptisine	y = 54 402 166x + 11 845	0.9992	0.00350	0.01051	193.53 ±3.59
sanguinarine	y = 79 920 166x + 40 324	0.9941	0.00756	0.02267	538.97 ±9.45
berberine	y = 87 636 814x - 27 662	0.9999	0.00050	0.00149	96.40 ±1.47
chelerythrine	y = 114 250 391x – 20 078	0.9998	0.00211	0.00632	506.85 ±6.10
